# Supplementary material for: The causal relationship between the human gut microbiota and pyogenic arthritis: a Mendelian randomization study
Source: Front Cell Infect Microbiol. 2024 Nov 26;14:1452480. doi: 10.3389/fcimb.2024.1452480 (PMC11629706; doi:10.3389/fcimb.2024.1452480)
Supplement: Supplementary file 1 [file DataSheet1.zip › mendelian test/ebi-a-GCST90027578.csv_leave_one_out.pdf]

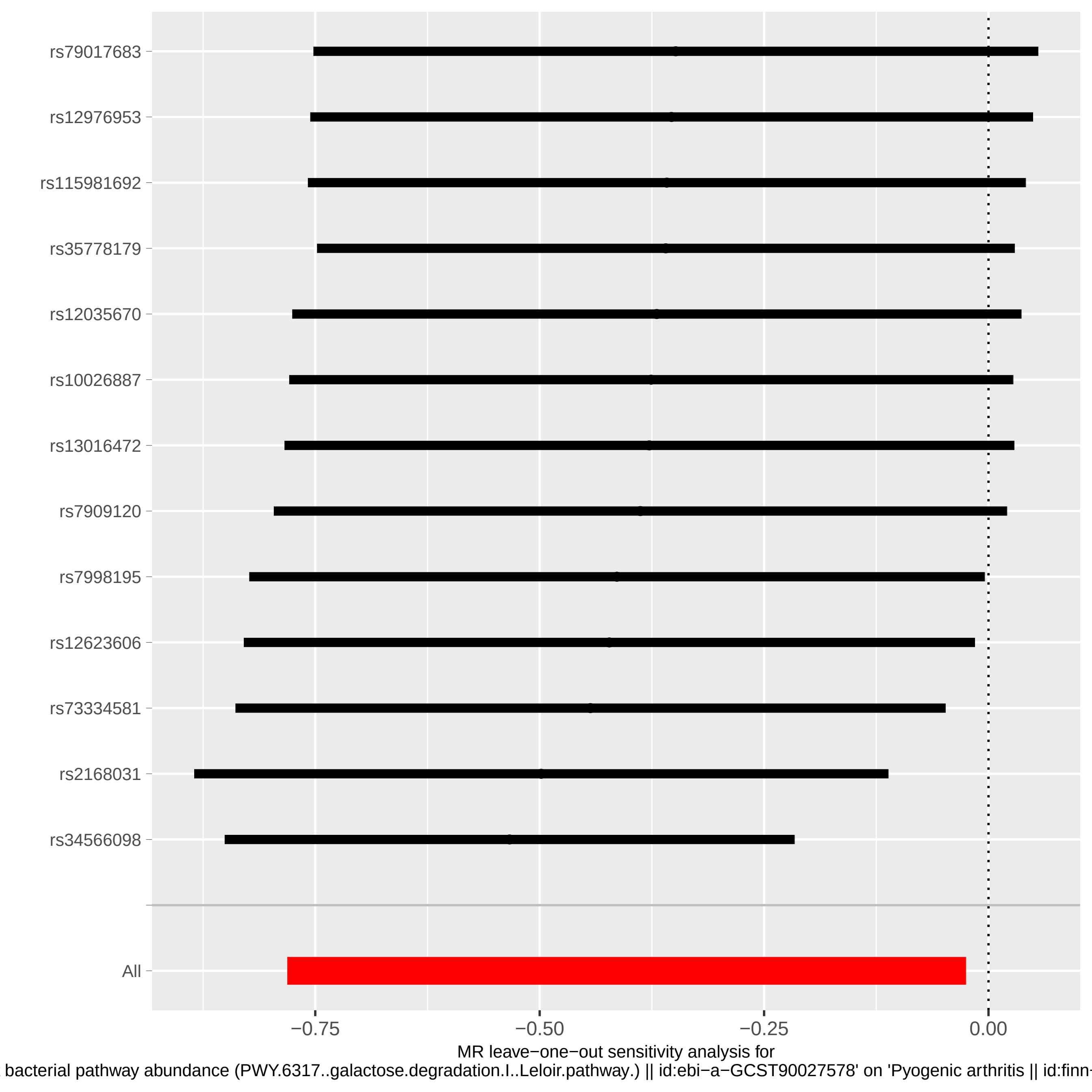

rs79017683
rs12976953
rs115981692
rs35778179
rs12035670
rs10026887
rs13016472
rs7909120
rs7998195
rs12623606
rs73334581
rs2168031
rs34566098
All
−0.75
−0.50
−0.25
0.00
MR leave−one−out sensitivity analysis for
bacterial pathway abundance (PWY.6317..galactose.degradation.I..Leloir.pathway.) || id:ebi−a−GCST90027578' on 'Pyogenic arthritis || id:finn-
